# Hyperthermia Using Antibody-Conjugated Magnetic Nanoparticles and Its Enhanced Effect with Cryptotanshinone

**DOI:** 10.3390/nano4020319

**Published:** 2014-04-23

**Authors:** Satoshi Ota, Naoya Yamazaki, Asahi Tomitaka, Tsutomu Yamada, Yasushi Takemura

**Affiliations:** 1 Department of Electrical and Computer Engineering, Yokohama National University, Yokohama 240-8501, Japan; E-Mails: ota-satoshi-gw@ynu.jp (S.O.); yamazaki-naoya-vr@ynu.ac.jp (N.Y.); yamada@ynu.ac.jp (T.Y.); 2 Department of Materials Science and Engineering, University of Washington, Seattle, WA 98195, USA; E-Mail: tomitaka@u.washington.edu

**Keywords:** magnetic nanoparticles, hyperthermia, antibody, apoptosis, cryptotanshinone

## Abstract

Heat dissipation by magnetic nanoparticles (MNPs) under an alternating magnetic field can be used to selectively treat cancer tissues. Antibodies conjugated to MNPs can enhance the therapeutic effects of hyperthermia by altering antibody-antigen interactions. Fe_3_O_4_ nanoparticles (primary diameter, 20–30 nm) coated with polyethylenimine (PEI) were prepared and conjugated with CH11, an anti-Fas monoclonal antibody. HeLa cell growth was then evaluated as a function of antibody and MNP/antibody complex doses. HeLa cell growth decreased with increased doses of the antibody and complexes. However, MNPs alone did not affect cell growth; thus, only the antibody affected cell growth. In hyperthermia experiments conducted using an alternating magnetic field frequency of 210 kHz, cell viability varied with the intensity of the applied alternating magnetic field, because the temperature increase of the culture medium with added complexes was dependent on magnetic field intensity. The HeLa cell death rate with added complexes was significantly greater as compared with that with MNPs alone. Cryptotanshinone, an anti-apoptotic factor blocker, was also added to cell cultures, which provided an additional anti-cancer cell effect. Thus, an anti-cancer cell effect using a combination of magnetic hyperthermia, an anti-Fas antibody and cryptotanshinone was established.

## 1. Introduction

Magnetic nanoparticles (MNPs) can be used in various medical fields as carriers for a drug delivery system (DDS), as contrast agents for magnetic resonance imaging and as heat sources for hyperthermia [1,2,3]. Magnetic capsules that encapsulate drugs and avoid loss due to elution in blood vessels have been synthesized as carriers for a DDS [4]. Synthesizing MNPs for use in a DDS and for hyperthermia has also been investigated. In particular, iron oxide nanoparticles, such as Fe_3_O_4_, have low cytotoxicity and have been investigated for their magnetic property with respect to the effects of their primary and secondary sizes, states and surface-modifying agents [5,6,7].

Hyperthermia is a less invasive method for cancer therapy, and tumor cells are more susceptible to heat than healthy cells. The clinical effects of hyperthermia using MNPs have been demonstrated for prostate cancer [8]. A magnetic field can be used to direct MNPs to a disease site to treat a deeply embedded tumor. MNPs are heated by applying an AC magnetic field of sufficient strength and frequency. Hyperthermia using MNPs is not restricted due to the unacceptable coincidental heating of healthy tissues, because MNPs can be used to selectively heat cancer tissues [2]. Tumor growth could be controlled in mouse C3H mammary carcinoma using hyperthermia treatment with superparamagnetic nanoparticles and excitation with an AC magnetic field [9]. Hyperthermia-induced apoptosis has been observed in human Raji cells, as confirmed by apoptosis-associated DNA fragmentation [10]. In addition, the integrative therapeutic and diagnostic application, called theranostics, has emerged. MNPs have been used both as a heat source for hyperthermia and as a contrast agent for magnetic resonance imaging (MRI) [11,12,13]. For *in vivo* applications, polyethylene glycol (PEG) is coated onto MNPs in order to avoid the reticuloendothelial system, due to opsonin absorbance onto MNPs and phagocytosis by macrophages [12,13].

It has been reported that an interactive therapy is synergistic, additive or antagonistic [14]. It is synergistic or additive when the effect of the combination is higher than each single effect or equal to each other, respectively. In contrast, it is antagonistic when the effect of the combination is lower than each single effect and non-interactive. The combined use of MNPs and antibodies increases these therapeutic effects. Antibody targeting of tumor-associated antigens (TAA) enhances the selective effects in cancer tissues [15]. Using G250 antibody-conjugated magnetoliposomes, MNPs encased in neutral liposomes were used to target renal cell carcinoma and were suitable for efficient hyperthermia treatment [16]. Ch11 is a monoclonal antibody directed against Fas, which is a cell surface protein that belongs to the tumor necrosis factor (TNF) receptor family and induces cellular apoptosis [17]. Apoptosis induced by anti-Fas antibodies is indistinguishable from the cytolytic activity of TNF [18]. Target cells undergo apoptosis when the Fas ligand (FasL) binds to Fas [17]. Fas stimulation induces both caspase-8-dependent and -independent activation of Bak, a pro-apoptotic member of the Bcl-2 family [19,20]. An anti-Fas antibody mimicked the function of FasL and induced target cells apoptosis [21,22]. It has also been shown that CH11 could induce HeLa cell apoptosis [20].

For this study, polyethylenimine (PEI)-coated Fe_3_O_4_ nanoparticles were prepared and then conjugated with CH11 antibodies. PEI modifications disperse MNPs due to cationic PEI charges and the antibody interfaces with MNPs. HeLa cell growth in the presence of MNPs, CH11 antibodies and MNP/antibody complexes was then evaluated. Cell growth as a function of antibody and complex dose was also assessed. For hyperthermia experiments using these complexes, cell viability was determined as a function of AC magnetic field intensity. In addition, cryptotanshinone, which induces anti-tumor activity, was added to cell cultures in conjunction with hyperthermia and antibody treatment. Cryptotanshinone, the major tanshinone isolated from *Salvia miltiorrhiza* Bunge, effectively blocks the expression of Bcl-2, an anti-apoptotic member of the Bcl-2 family, and promotes cellular apoptosis [23].

## 2. Results and Discussion

### 2.1. HeLa Cell Growth in the Presence of CH11 Antibody or MNP/Antibody Complexes

**Figure 1:**
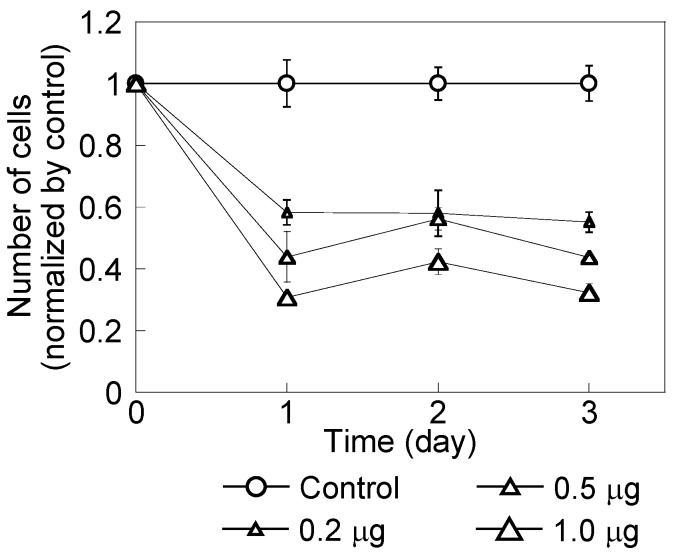
HeLa cell growth in the presence of a CH11 antibody added at 0.2, 0.5 and 1.0 μg/mL. The control did not include this antibody. Cell numbers were normalized by the number of control cells. Cell numbers decreased with increased antibody dose.

**Figure 2:**
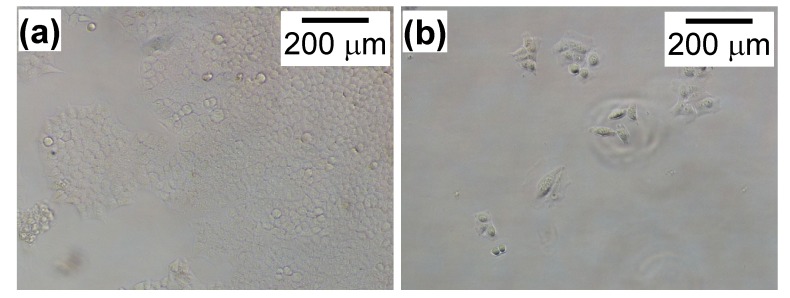
Images of HeLa cells without (**a**) and with (**b**) a CH11 antibody at 1.0 μg/mL. There were fewer cells after adding this antibody compared with those without this antibody.

Figure 1 shows HeLa cell growth over three days after adding a CH11 antibody. Cell numbers were normalized by the number of cells in the absence of antibody. HeLa cell growth was dependent on antibody dose. After one day in the presence of CH11 antibodies, cell growth was reduced by 60% or more. With 1.0 μg/mL of antibody, cell growth was 30% of the control. At three days, cells with 1.0 μg/mL of antibody lost their ability to form colonies (Figure 2). This indicated that the CH11 antibody induced cellular apoptosis [20,24].

**Figure 3:**
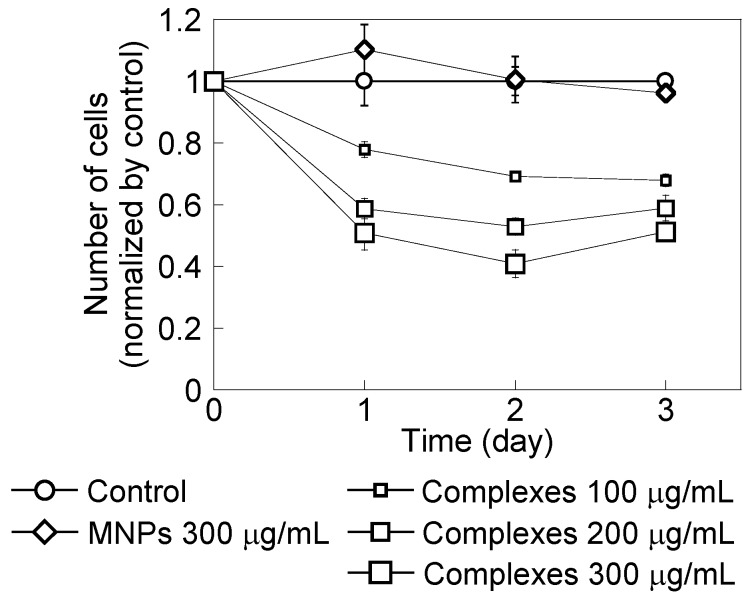
HeLa cell growth in the presence of polyethylenimine (PEI)-coated magnetic nanoparticles (MNPs) at 300 μg/mL and with MNP/antibody complexes added at 100, 200 and 300 μg/mL. The control did not include these treatments. Cell numbers were normalized by the number of control cells. Cell numbers decreased with increasing complex doses.

**Figure 4:**
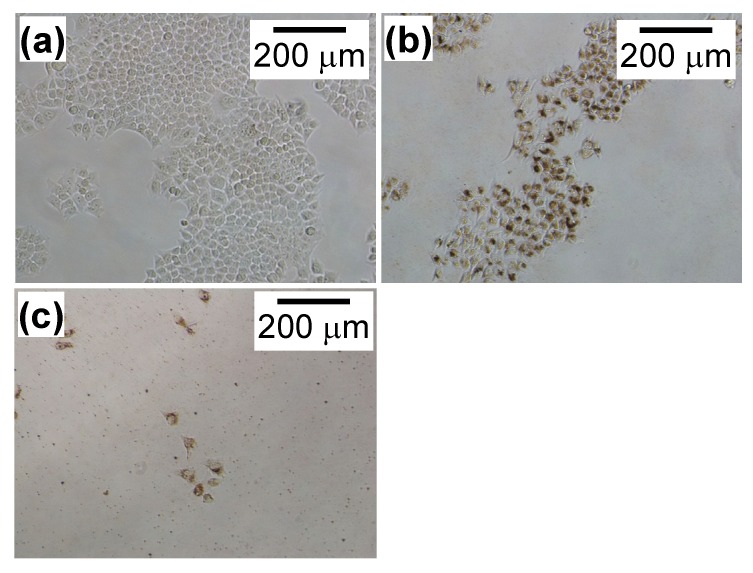
Images of HeLa cells with (**b**) PEI-coated MNPs added at 300 μg/mL or (**c**) MNP/antibody complexes added at 300 μg/mL. The image in (**a**) is of the control cells in Figure 1. There were fewer cells after adding PEI-coated MNPs and complexes compared to without these treatments.

Figure 3 shows HeLa cell growth in the presence of PEI-coated MNPs or MNP/antibody complexes for three days. Cell numbers were normalized to the number of cells without MNPs and complexes. PEI-coated MNPs did not affect cell growth. However, cell growth decreased with an increased dose of these complexes. Figure 4 shows that cells with added complexes lost their ability to form colonies compared with the control and when PEI-coated MNPs were added.

**Figure 5:**
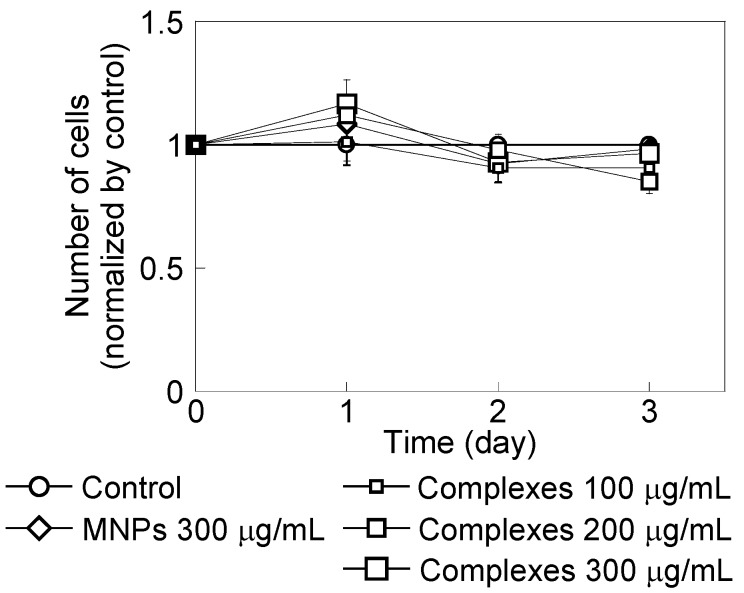
TIG-1 cell growth in the presence of PEI-coated MNPs at 300 μg/mL and with MNP/antibody complexes at 100, 200 and 300 μg/mL. The control did not include these treatments. Cell numbers were normalized to the number of control cells.

Figure 5 shows TIG-1 cell (human lung fibroblast line) growth in the presence of PEI-coated MNPs or the complexes for three days. Cell numbers were normalized to the number of cells without MNPs and complexes. For TIG-1 cells, these complexes only minimally affected cell growth, because the antibody used selectively targeted TAA [15].

### 2.2. Effect of Magnetic Hyperthermia Combined with CH11 Antibodies

**Figure 6:**
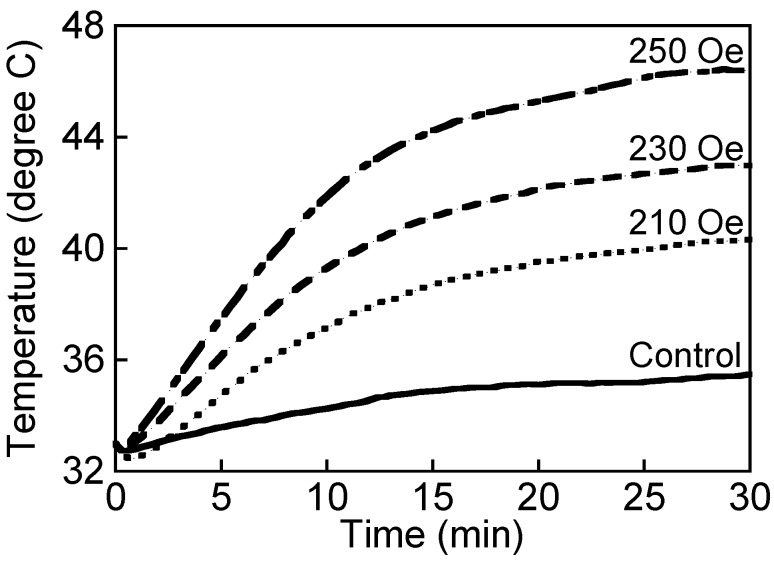
The temperature rise of culture medium added with PEI-coated MNPs of 200 μg/mL in the case of applying an AC magnetic field for 30 min. The magnetic field intensity was 210, 230 and 250 Oe. The frequency of magnetic field was 210 kHz. The control indicates the sample added with nothing in the case of applying the AC magnetic field of 250 Oe. MNPs were effective in hyperthermia. The temperature rise depended on AC magnetic field intensity.

The temperature increase in the culture medium was dependent on the magnetic field intensity used (Figure 6). The temperature of the medium after applying the magnetic fields of 210, 230 and 250 Oe for 30 min was 40.3, 42.8 and 46.2 °C, respectively. When applying an AC magnetic field of >230 Oe, the temperature was >42.0 °C. The medium that contained MNPs in a 35-mm dish was measured using a fiber-optic thermometer while applying an AC magnetic field. Temperatures of >42.0 °C can cause sublethal damage to HeLa cells [25]. Conjugating CH11 antibodies to PEI-coated MNPs did not influence the increase in temperature.

**Figure 7:**
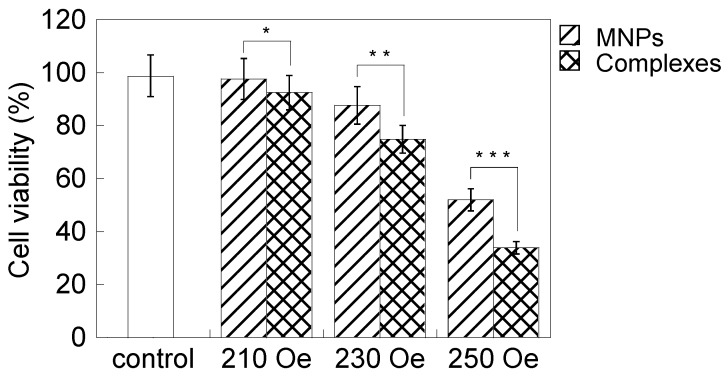
HeLa cell viability with added PEI-coated MNPs or MNP/antibody complexes and the application of an AC magnetic field for 1 h. The magnetic field intensities were 210, 230 and 250 Oe. The AC magnetic field frequency was 210 kHz. The control did not include MNPs or complexes while applying an AC magnetic field of 250 Oe. Cell viability decreased with increased magnetic field intensity. The complexes decreased cell viability compared with PEI-coated MNPs. The confirmation of cell viability decrease by adding the complexes in each magnetic field is marked with an asterisk (*p* < 0.05).

**Figure 8:**
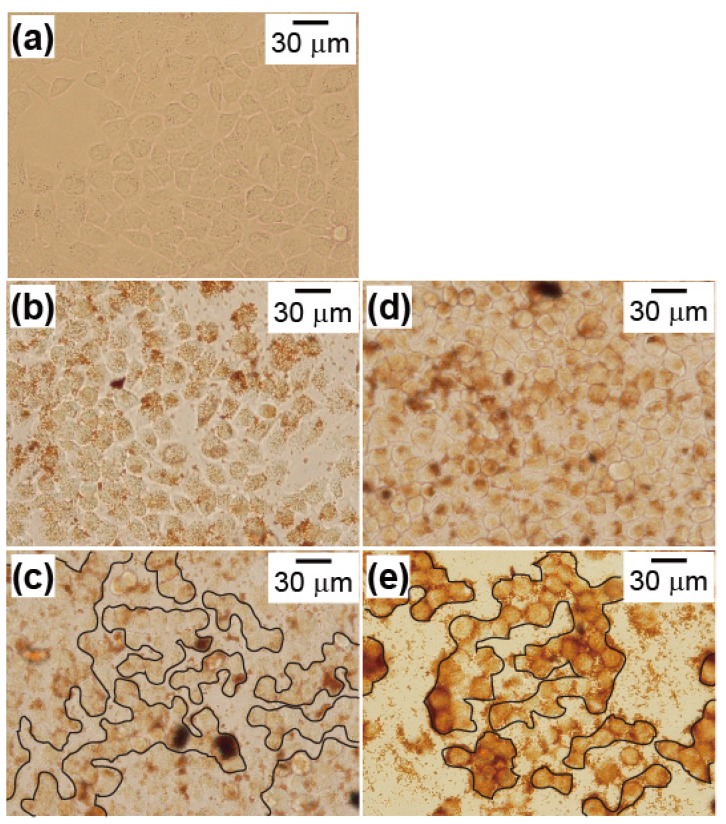
Images of HeLa cells in the presence of PEI-coated MNPs or MNP/antibody complexes and the application of an AC magnetic field for 1 h. Cell conditions and magnetic field intensities were: (**b**) PEI-coated MNPs and 210 Oe; (**c**) PEI-coated MNPs and 250 Oe; (**d**) complexes and 210 Oe; and (**e**) complexes and 250 Oe. The image in (**a**) is of the control cells in Figure 7. The AC magnetic field frequency was 210 kHz. The clusters in (**c**) and (**e**) are cell colonies. Cells had adhered and formed large colonies in (**a**), (**b**) and (**d**). However, cells were divided into a number of small colonies in (**c**) and (**e**).

Figure 7 shows cell viability results when adding PEI-coated MNPs or the complexes and applying an AC magnetic field for 1 h. The decrease in cell viability was minimal with a magnetic field intensity of 210 Oe, because the temperature of the heat generated was <42.0 °C. However, cell viability decreased with a magnetic field intensity of 230 Oe, and the decrease was significant with 250 Oe. In particular, cell death was significant when adding the complexes and applying AC magnetic field intensities of 230 and 250 Oe. This indicated that this antibody had enhanced the therapeutic effect of hyperthermia, because of its selective anti-tumor effect by interacting with TAA [15]. However, it was not examined if this anti-cancer effect was induced by the increase of the hyperthermic effect, due to the cancer-targeting effect of the complexes in this study. PEI-coated MNPs accumulated on the cell surface without antibody. Accumulation of PEI-coated MNPs was observed by phase contrast microscopy. Figure 8 shows that those cells with reduced viability in Figure 7 had lost their ability to form colonies. The combined effect of magnetic hyperthermia with this antibody indicated in Figure 7 was in good agreement with the images shown in Figure 8. It was not examined and discussed whether this anti-cancer effect derived from apoptosis or necrosis.

### 2.3. Cryptotanshinone-Induced Cellular Apoptosis

**Figure 9:**
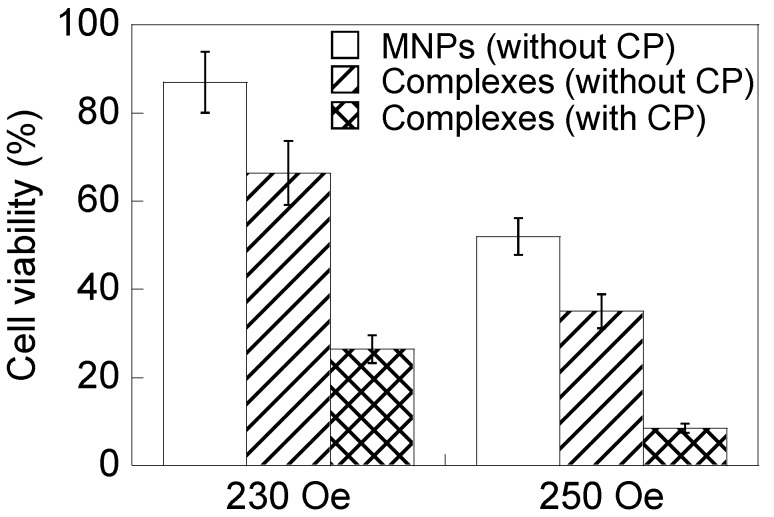
HeLa cell viability in the presence of PEI-coated MNPs, MNP/antibody complexes or MNP/antibody complexes and cryptotanshinone (CP) and when applying an AC magnetic field for 1 h. The magnetic field intensities were 230 and 250 Oe. The AC magnetic field frequency was 210 kHz. Cryptotanshinone effectively reduced cell viability.

Cell viability was significantly reduced with the combination of cryptotanshinone with hyperthermia and antibody compared with the hyperthermia treatment combined with antibody (Figure 9). Bcl-2 is expressed in response to apoptotic stimuli, such as irradiation and anti-cancer drugs [19]. Cytochrome c release from mitochondria is downregulated by Bcl-2 [26]. Cytochrome c induces caspase-mediated apoptosis [27]. Cryptotanshinone inhibits Bcl-2 expression by inhibiting mitogen-activated protein kinase (MAPK) activity, and apoptosis is promoted by the unregulated release of cytochrome c [23,27]. It was found that the cell growth and cell number were not affected by cryptotanshinone. Cryptotanshinone itself does not promote cell death, as its effect is limited to controlling the expression of anti-apoptotic proteins. Cell survival declined to 12% when the complexes along with cryptotanshinone were added and a magnetic field intensity of 250 Oe (culture medium temperature, 46.2 °C) was used. This showed that cryptotanshinone significantly contributed to the anti-cancer cell effects of magnetic hyperthermia and antibody treatment.

## 3. Experimental Section

### 3.1. Materials and Reagents

Fe_3_O_4_ nanoparticles (primary diameter, 20–30 nm) were obtained from Nanostructured and Amorphous Materials, Inc. Polyethylenimine max (PEI; mw 40,000) was from Polysciences, Inc. Monoclonal antibody CH11, *N*-succinimidyl 3-(2-pyridyldithio)propionate (SPDP), 2-iminothiolane hydrochloride and cryptotanshinone were from Immunotech S.A. (Marseille, Freance), Pierce Thermo Scientific K.K. (Rockford, IL, USA), Nacalai Tesque (Kyoto, Japan) and Carbosynth Ltd. (Berkshire, UK), respectively. SPDP and 2-iminothiolane hydrochloride were used to modify PEI and CH11, respectively. SPDP is a cross-linking agent that conjugates amino groups with thiol groups through disulfide bonds. To conjugate an antibody to PEI-coated MNPs, PEI amino groups on the surfaces of MNPs were modified using SPDP. Antibody amino groups were modified using 2-iminothiolane hydrochloride and substituted with thiol groups.

### 3.2. Surface Coating and Synthesis of MNP/Antibody Complexes

Fe_3_O_4_ nanoparticles (100 mg) were dispersed in 25 mL of a PEI solution (0.2 mg/mL) by supersonification for 10 min. This solution was then centrifuged at 743 × *g* (*R* = 7.39 cm) for 15 min. The supernatant was centrifuged at 10,000 × *g* (*R* = 8.8 cm) for 30 min, and the precipitate was collected as PEI-coated Fe_3_O_4_ nanoparticles.

PEI-coated Fe_3_O_4_ nanoparticles were dispersed in 1 mL of phosphate-buffered saline (PBS), mixed with 25 μL of an SPDP solution [SPDP at 20 mM in Dimethyl sulfoxide (DMSO)] for 30 min and then centrifuged at 10,000 × *g* (*R* = 8.8 cm) at room temperature for 30 min. The precipitate was collected as SPDP-modified PEI-coated Fe_3_O_4_ nanoparticles. These were dispersed in 1 mL of PBS.

**Figure 10:**
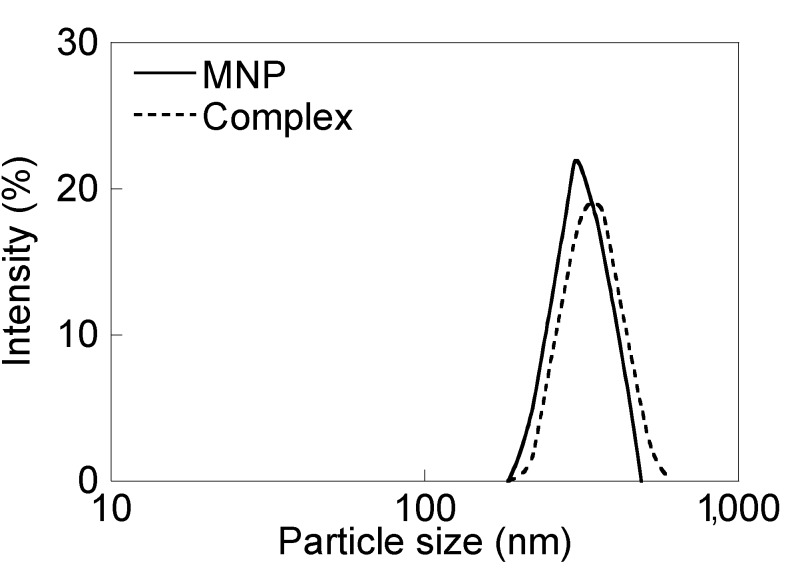
The size distribution of PEI-coated Fe_3_O_4_ nanoparticles and the complexes in phosphate-buffered saline (PBS). The size of PEI-coated Fe_3_O_4_ nanoparticles and the complexes were 318 ± 57 nm and 349 ± 70 nm, respectively.

The CH11 antibody (50 μg) was dissolved in 1 mL of PBS, and 18 μL of a 2-iminothiolane hydrochloride solution (2 mg/mL) was added at room temperature for 1 h. SPDP-modified PEI-coated Fe_3_O_4_ nanoparticles were mixed with 2-iminothiolane hydrochloride modified CH11 at room temperature for 12 h. This solution was then centrifuged at 10,000 × *g* (*R* = 8.8 cm) at room temperature for 30 min. The precipitate was collected as CH11-conjugated Fe_3_O_4_ nanoparticles (MNP/antibody complexes). Conjugation of Fe_3_O_4_ nanoparticles with antibody was confirmed by the BCA protein assay. The size distribution of PEI-coated Fe_3_O_4_ nanoparticles and the complexes in (PBS) are shown in Figure 10. Comparing the results in Figure 3 with those in Figure 1, the cell growth inhibitory effect of the antibody at 0.2 μg/mL was confirmed using the complexes at 200 μg/mL. The number of molecules of the antibody bound to a MNP was calculated to 1.2 × 10^3^ by the above result and the size of PEI-coated MNPs dispersed in PBS.

### 3.3. Cell Culture

HeLa cells (human cervical carcinoma line) and TIG-1 cells (human lung fibroblast line) were cultured in Dulbecco’s modified Eagle medium (DMEM) supplemented with 10% fetal bovine serum (FBS) and 1% penicillin-streptomycin (PS).

### 3.4. Cell Growth Inhibition

HeLa cells and TIG-1 cells were cultured in a 24-well plate at a density of 10,000 cells/well for 24 h. For experimental treatments, the following were also added to the wells: CH11 antibody at 0.2, 0.5 or 1.0 μg/mL; PEI-coated MNPs at 300 μg/mL; or MNP/antibody complexes at 100, 200 or 300 μg/mL. Cell numbers were counted every day for 3 days after these different treatments.

### 3.5. Hyperthermia and Cryptotanshinone Treatment

To evaluate cell viability after hyperthermia treatment, cells were cultured in 35-mm dishes at a density of 50,000 cells/well for 24 h. The culture medium that contained PEI-coated MNPs or complexes at 200 μg/mL was prepared at 37 °C. This medium (2.5 mL) was added to a cell culture. One day after adding MNPs to the cell culture, an AC magnetic field was applied for 1 h. The magnetic field intensities used were 210, 230 and 250 Oe, and the AC magnetic field frequency was 210 kHz. Cells were incubated for 24 h after heating. Cell viability was evaluated by trypan blue exclusion. For combinations of hyperthermia using MNP/antibody complexes and cryptotanshinone, cryptotanshinone was added to the culture medium at a final concentration of 0.3 mg/mL. The medium with cryptotanshinone (2.5 mL) was added to cells for 15 min before adding PEI-coated MNPs and the complexes. When using these combinations with cryptotanshinone, the AC magnetic field intensities used were 230 and 250 Oe. In addition, both intracellular and extracellular PEI-coated MNPs were confirmed by Prussian blue staining. A smaller number of intracellular MNPs in cytoplasm were observed than extracellular MNPs on the cell surface. This probably indicated that the large number of MNPs were not internalized into cells, due to aggregation. Aggregation was induced by high concentration of MNPs.

## 4. Conclusions

The anti-cancer effects of magnetic hyperthermia combined with a CH11 antibody and cryptotanshinone were assessed. HeLa cell growth was reduced after treatment with this antibody and MNP/antibody complexes. Hyperthermia generated using PEI-coated MNPs and the application of an AC magnetic field resulted in effective cell death when the magnetic field intensity induced a sufficient temperature increase for killing cancer cells. In addition, hyperthermia treatment using MNP/antibody complexes was quite effective for cancer cell treatment. Moreover, adding cryptotanshinone further reduced HeLa cell survival. Thus, the combination of magnetic hyperthermia, an antibody (CH11; anti-Fas) and cryptotanshinone resulted in a significant anti-cancer cell effect.
